# Circulation of Reassortant Influenza A(H7N9) Viruses in Poultry and Humans, Guangdong Province, China, 2013

**DOI:** 10.3201/eid2012.140765

**Published:** 2014-12

**Authors:** Changwen Ke, Jing Lu, Jie Wu, Dawei Guan, Lirong Zou, Tie Song, Lina Yi, Xianqiao Zeng, Lijun Liang, Hanzhong Ni, Min Kang, Xin Zhang, Haojie Zhong, Jianfeng He, Jinyan Lin, Derek Smith, David Burke, Ron A.M. Fouchier, Marion Koopmans, Yonghui Zhang

**Affiliations:** Guangdong Provincial Center for Disease Control and Prevention, Guangzhou, China (C. Ke, J. Lu, J. Wu, D. Guan, L. Zou, T. Song, L. Yi, X. Zeng, L. Liang, H. Ni, M. Kang, X. Zhang, H. Zhong, J. He, J. Lin, Y. Zhang);; University of Cambridge, Cambridge, UK (D. Smith, D. Burke);; Erasmus MC, Rotterdam, the Netherlands (R.A.M. Fouchier, M. Koopmans);; National Institute of Public Health and the Environment, Bilthoven, the Netherlands (M. Koopmans)

**Keywords:** influenza, H7N9, H9N2, emergence, reassortment, human, poultry, influenza A, H3N2, viruses, respiratory infections, Guangdong Province, China

## Abstract

Influenza A(H7N9) virus emerged in eastern China in February 2013 and continues to circulate in this region, but its ecology is poorly understood. In April 2013, the Guangdong Provincial Center for Disease Control and Prevention (CDC) implemented environmental and human syndromic surveillance for the virus. Environmental samples from poultry markets in 21 city CDCs (n = 8,942) and respiratory samples from persons with influenza-like illness or pneumonia (n = 32,342) were tested; viruses isolated from 6 environmental samples and 16 patients were sequenced. Sequence analysis showed co-circulation of 4 influenza A(H7N9) virus strains that evolved by reassortment with avian influenza A(H9N2) viruses circulating in this region. In addition, an increase in human cases starting in late 2013 coincided with an increase in influenza A H7 virus isolates detected by environmental surveillance. Co-circulation of multiple avian influenza viruses that can infect humans highlights the need for increased surveillance of poultry and potential environmental sources.

Human infection with a novel avian-origin influenza A(H7N9) virus was first identified in eastern China in February 2013, and a major outbreak occurred from the end of March through the beginning of May (*1*,*2*). Risk factors identified for a severe course of illness were older age (>65 years) and underlying illnesses, including hypertension and chronic lung diseases, but the full spectrum of illness associated with H7N9 virus remains to be determined (*3*). Molecular characterization has shown that the H7N9 virus emerged by reassortment between H7, N9, and H9N2 avian influenza viruses from the Yangzi River Delta region; this reassortment likely occurred in eastern China in early 2012 (*2*,*4*,*5*). Because infection with H7N9 virus does not cause overt disease in poultry, the spread of infection can be insidious, and the exact modes and extent of geographic spread of the virus remain unknown. Birds commonly used for egg and meat production in China differ in their susceptibility, levels of virus shedding, and ability to transmit the virus; data from experimental infections indicate that quail and chickens are possible candidates for virus transmission (*6*). Detailed analysis of viruses from human patients showed evidence for diversification through reassortment of the originally detected viruses with strains from poultry in the same geographic region (*7*). Infection of humans is linked to exposure to poultry or to environments where poultry are present; closure of live poultry markets (LPMs) has had a measurable effect on controlling the spread of infection (*8*,*9*).

After H7N9 virus was initially detected in eastern China, the Guangdong Provincial Center for Disease Control and Prevention (CDC) implemented environmental surveillance and syndromic surveillance in humans. After H7N9 virus infection in humans was reported, enhanced sentinel hospital surveillance and environmental sampling programs were implemented to identify possible cases and analyze the evolution of the virus. The first case of human infection with H7N9 virus in Guangdong was confirmed in a patient with severe pneumonia on August 10, 2013. This case-patient was a 51-year-old female poultry worker at a local LPM in Huizhou city in Guangdong Province. No other cases were reported until October 2013, when a wave of cases began in several provinces, including Guangdong. We present results of ongoing environmental and human syndromic surveillance for H7N9 virus circulation in Guangdong Province, as well as virologic analyses related to the natural history of this outbreak. 

## Materials and Methods

### Syndromic Surveillance in Humans

Since April 16, 2013, enhanced surveillance for influenza A(H7N9) virus has been conducted in 28 sentinel hospitals and 23 collaborating laboratories in 21 cities in Guangdong Province, China. All specimens are first tested for avian influenza A virus; specimens with positive results are then further tested in the laboratories of the 21 city CDCs for subtypes H5, H7, and H9 by using commercial real-time reverse transcription PCR (RT-PCR) (Liferiver, Shanghai ZJ Bio-Tech, Shanghai, China). Results are further verified at the Guangdong Provincial CDC. H7-positive specimens are tested for N9 gene segments by using real-time RT-PCR (*2*). H7N9-positive swab materials and sputum samples are then blindly passaged for 2 or 3 generations in 9- or 10-day-old embryonated chicken eggs for virus isolation. Hemagglutination-positive allantoic fluids are collected, and viruses are subtyped by hemagglutination and neuraminidase inhibition (HI and NI) assays by using a panel of reference antiserum, as described by Huang et al. (*10*). Standard precautions are taken to avoid cross-contamination of specimens. All H7 influenza viruses isolated from patients undergo complete genome sequencing.

### Environmental Surveillance

In April 2013, the Guangdong Provincial CDC launched an environmental surveillance program to monitor for avian influenza viruses in LPMs. Environmental samples were collected from LPMs in Guangdong Province during April 15, 2013–February 28, 2014. Each week during April 15–May 31, 2013, twenty environmental samples per market were collected from selected markets in 21 cities at the prefecture level. The environmental sampling was done by collecting wet-swab specimens from poultry feces, chicken epilator surfaces, chopping board surfaces, cage surfaces, and sewage. If a human H7N9 case was confirmed and the case-patient had exposure in an LPM, >20 environmental samples would be collected from that market.

Samples were collected from poultry feces by swabbing the surfaces of the chicken epilator, chopping boards, and cages 4–8 times with separate cotton-tipped swabs (Copan Italia, Brescia, Italy). The swabs were then inserted into a tube containing 3 mL of virus transport medium (Copan Italia) and stored at 4°C before shipping to the CDC laboratory. At arrival in the laboratory, samples were vortexed and swabs were discarded. Total RNA and DNA were extracted simultaneously from 200 μL supernatant of the sample by using the QIAamp minElute Virus Spin Kit (QIAGEN, Crawley, UK), according to the manufacturer’s instructions. Then, 5 μL of eluate was tested for influenza A; thereafter, testing for subtypes H5, H7, and H9 was done by commercial real-time reverse transcription PCR (RT-PCR) (Liferiver, Shanghai ZJ Bio-Tech). H7-positive specimens were further subjected to N9 gene-specific RT-PCR. Dual positive samples were blindly passaged for 2–3 generations in 9- to 10-day-old embryonated chicken eggs for virus isolation. Hemagglutinin (HA)–positive isolates were collected and further subtyped by HI and NI assays using a panel of reference antiserum, as described by Huang et al. (*10*). Standard precautions were taken to avoid cross-contamination of specimens. Monoinfluenza virus infection (without mixed infection of other subtypes) was further confirmed by RT-PCR before whole-genome sequencing. All H7N9 isolates from LPMs were subjected to complete genome sequencing, as were 7 H9 influenza viruses that were isolated from different LPMs during May–December 2013.

### Whole-Genome Deep Sequencing

For all selected isolates, all 8 gene segments (HA, neuraminidase [NA], nucleoprotein [NP], polymerase basic 1 [PB1] and 2 [PB2], polymerase acidic [PA], matrix [M], and nonstructural [NS]) were sequenced by using a next-generation sequencing strategy for influenza A virus sequencing with the Ion PGM System and PathAmp FluA Reagents (Life Technologies, Carlsbad, CA, USA). Viral RNA extraction was performed by using the QIAamp Viral RNA Mini Kit (QIAGEN, Hilden, Germany). Reverse transcription PCR amplification of all 8 gene segments were performed by using PathAmp Flu A Preamplification Reagents (Life Technologies). Amplicons were purified and quantitated by using the Ampure XP purification kit (Beckman, Brea, CA, USA). The genomic libraries were prepared with the Ion Xpress Plus Fragment Library Kit (Life Technologies). Enzymatic fragmentation was used for the 200-bp read protocol with a 10-min incubation time. Samples were assigned barcodes by using the Ion Xpress Barcode Adapters 1–32 Kit (Life Technologies). Automated template preparation was performed by using the Ion OneTouch 2 System (Ion PGM Template OT2 200 Kit; Life Technologies). Final sequencing was performed with the Ion PGM Sequencing 200 Kit v2 (Life Technologies) using the Ion 316 Chip V2.

### Genome Sequence Alignment

Genome sequence assembly was done with the pathogen analysis program of the Ion PGM server (Life Technologies). Multiple sequence alignment against previously published complete genome sequences of H7N9 and H9N2 viruses was performed by using ClustalW (http://www.clustal.org) and BioEdit software version 7.14 (http://www.mbio.ncsu.edu/bioedit/bioedit.html). The whole-genome sequences were submitted to GISAID (http://www.gisaid.org; accession nos. EPI148417 and EPI151429–151437).

### Phylogenetic Analysis and Data Visualization

Maximum-likelihood trees were estimated for all 8 gene segments by using MEGA version 5.2 (http://www.megasoftware.net) with the general time reversible + Γ model (*11*). To assess the robustness of individual nodes on phylogenetic trees, a bootstrap resampling process (1,000 replications) and the neighbor-joining method were used.

The initial trees were constructed in PhyML by using general time reversible + I + Γ4 as the evolutionary model (*12*). GARLI (https://code.google.com/p/garli/) was run on the best tree from PhyML for 1 million generations to optimize tree topology and branch lengths.

## Results

### Syndromic Surveillance

A total of 34,342 samples from patients with pneumonia or influenza-like illness from 21 cities in Guangdong Province were tested during April 2013–February 2014. Of these, samples from 81 patients from 10 cities tested positive for influenza A(H7N9) virus. With the exception of 1 case confirmed in August 2013, all other cases were found starting in October 2013. In total, 6 cases were confirmed in 2013 and 75 in early 2014. 

Patient characteristics are summarized in Table 1. Most patients were male, and the median age of all patients was 52 years. Of the 81 patients, 70 (84%) had severe illness, which likely reflected the design of the surveillance (hospitalized patients). Twenty-two (27%) patients died; 14 remained hospitalized as of May 2014, and 45 had recovered. Overall, 73 patients (90.1%) reported poultry contact (Table 1).

**Table 1:** Demographic and clinical characteristics of influenza A(H7N9) case-patients detected through enhanced surveillance in Guangdong Province, China, March 2013–February 2014

Characteristic	No. (%) case-patients
Sex*	
M	48 (59.3)
F	33 (40.7)
Age group, y†	
≤20	12 (14.8)
21–40	13 (16.0)
41–60	25 (30.9)
≥61	31 (38.3)
Exposure history	
Live poultry market visit	47 (58.0)
Household poultry breeding	10 (12.3)
Poultry processing	5 (6.2)
Mixed	11 (13.6)
None known	8 (9.9)
Clinical use of oseltamivir‡	
Used	65 (80.2)
Unknown	16 (19.8)
Clinical condition	
Influenza-like illness	11 (13.6)
Pneumonia	14 (17.3)
Mild influenza	2 (2.5)
Severe influenza	54 (66.7)
Outcome	
Death	22 (27.2)
Recovery	45 (55.6)
Hospitalization	14 (17.3)

*M:F sex ratio 1.5:1. †Median patient age 52.0 y. ‡Mean time from symptom onset to diagnosis 8.5 d; mean time from symptom onset to use of oseltamivir 5.2 d.

### Environmental Surveillance

**Figure 1:**
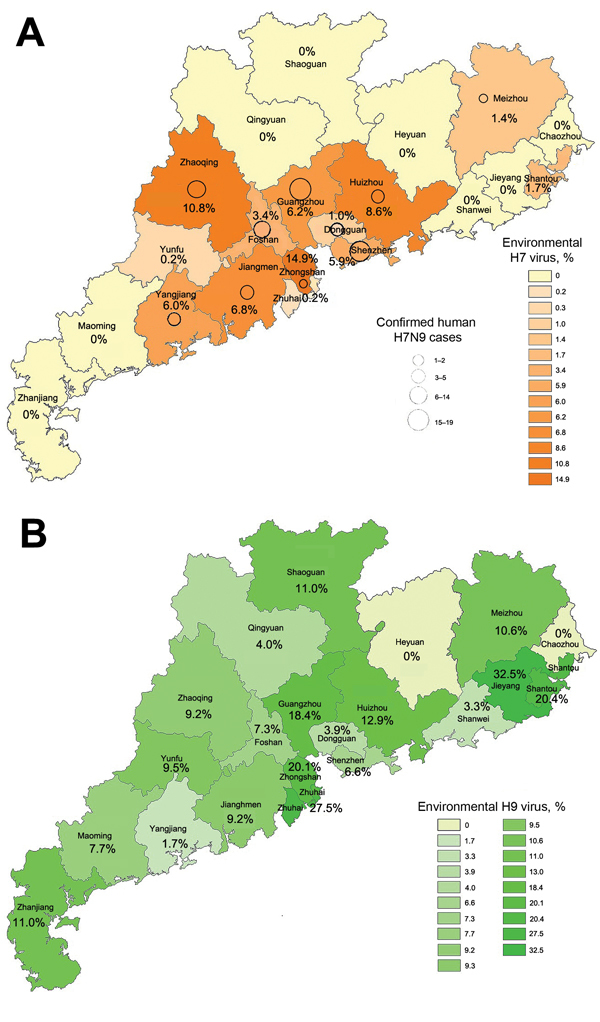
Distribution of influenza A H9 (A) and H7 (B) viruses, Guandong Province, China, 2013–2014. Shading indicates percentage of environmental swab specimens from live poultry markets in each region that were positive for each influenza subtype by reverse transcription PCR. Circles indicate locations of human cases; larger circles indicate higher numbers of cases.

A total of 8,942 swab samples from LPMs were collected and screened in laboratories of city CDCs at the prefecture level and at Guangdong Provincial CDC (*2*). Overall, 425 samples (4.8%) from 13 cities tested positive for H7 viruses, and 1,050 (11.8%) from 19 cities were positive for H9 viruses. The prevalence of viral genes in regions with positive findings ranged from 1%–14% of swab samples for H7 viruses and 1.7%–27.5% for H9. The H9 detection rate remained ≈12% throughout the year, except during June, when only 4% of samples tested positive. H9 viruses were detected throughout Guangdong Province (19 of the 21 cities; Figure 1, panel A). In contrast, only 1 H7-virus positive specimen was detected during March–September 2013 (in Meizhou in April), but detections of H7 viruses increased beginning in October: from 1 site in October, 3 in November, 8 in January, and 7 in February. H7 viruses were detected in the Pearl River Regions of Guangdong Province (Figure 1, panel B). Human cases were found in 10 of the 13 cities where environmental samples were positive for H7, and in 0 of the 8 cities where no H7 viruses were found in environmental samples (Figure 1).

### Sequence Analysis

#### Internal Genes

**Figure 2:**
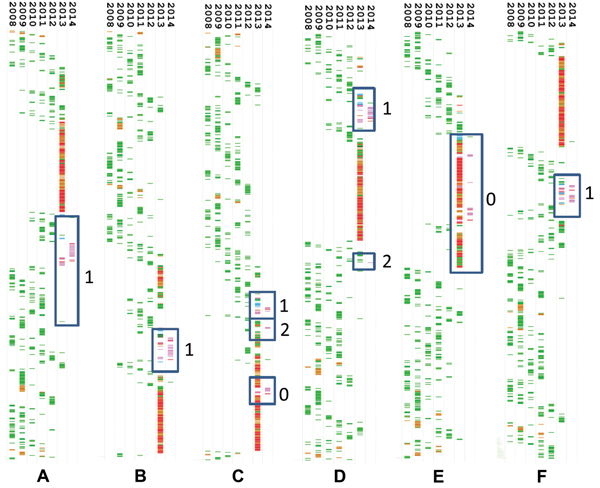
Timed phylogenies of internal gene segments of influenza A(H7N9) viruses detected in 16 patients from Guangdong Province, China, 2008–2014. A) Polymerase basic 2; B) polymerase basic 1; C) polymerase acidic; D) nucleoprotein; E) matrix; F) nonstructural. Avian H9N2 viruses are shown in green, avian H9N2 viruses from Guangdong Province in blue, human H7N9 sequences from Guangdong Province in pink, and human H7N9 sequences from the main case cluster in 2013 in eastern China in red. Blue boxes indicate clusters of patient sequences on separate branches; numbers correspond with the reassortant numbers listed in Table 2, where 0 indicates original genes from the 2013 cluster of influenza A(H7N9). Individual trees are shown in the Technical Appendix.

Whole-genome sequences were determined for the H7N9 virus isolates from 16 patients and 6 environmental samples and for the 9 H9 viruses collected during environmental surveillance. Phylogenetic trees were constructed for each internal gene segment against all currently available H7N9 and H9N2 virus sequences from the National Center for Biotechnology Information and GISAID. Phylogenetic analysis of the whole-genome sequences showed the clear separation of PB2, PB1, and NS gene sequences of the Guangdong patient isolates from those detected from patients from eastern China (Figure 2).

Gene sequences on the same branch were reviewed to identify region of origin; these sequences matched internal genes from H9N2 viruses detected in southern China (Technical Appendix). M genes of all viruses clustered with the genes detected in viruses from the main H7N9 virus cluster in 2013, whereas diversity was seen in the PA (main 2013 variant and 2 additional lineages) and NP (2 lineages) genes. Combined, the H7N9 virus isolates that were sequenced appeared to represent at least 4 different reassortants (Table 2).

**Table 2:** Genomes of influenza A(H7N9) viruses detected in 16 patients and from environmental samples in Guangdong Province, China*

Sample identification	PB2 variant	PB1 variant	PA variant	NP variant	M variant	NS variant
Patient samples						
HZ-Y/01/2013	1	1	0	1	0	1
FS-Y/019/2014	1	1	0	1	0	1
FS2-Y/033/2014	1	1	0	1	0	1
FS2-T/034/2014	1	1	0	1	0	1
SZ-T/035/2014	1	1	0	1	0	1
SZ-Y/036/2014	1	1	0	1	0	1
A/Hong Kong/734/2014	1	1	0	1	0	1
DG-Y/02/2013	1	1	1	1	0	1
DG-T/03/2013	1	1	1	1	0	1
YJ-T/04/2013	1	1	1	1	0	1
YJ-T/05/2013	1	1	1	1	0	1
YJ2-T/012/2014	1	1	1	1	0	1
YJ2-Y/013/2014	1	1	1	1	0	1
FS-Y/029/2014	1	1	1	1	0	1
Hong Kong 2212982	1	1	1	1	0	1
GZ-T/010/2014	1	1	2	1	0	1
FS-Y/031/2014	1	1	2	1	0	1
SZ-T/026/2014	1	1	0	2	0	1
Environmental samples						
Guangdong/02620/2014	1	1	0	1	0	1
Guangdong/24997/2014	1	1	0	1	0	1
Guangdong/0092/2014	1	1	1	1	0	1
Guangdong/02124/2014	1	1	1	1	0	1
Guangdong/25003/2014	1	1	1	1	x	1
Guangdong/02125/2014	1	1	2	1	0	1
Variant origin	SC	SC	PA-1: SC	NP-1: SC		SC
			PA-2: EC	NP-2:EC		

*Lineages shown correlated with those shown Figure 2. Shading indicates grouping of 4 reassortant types. Variant origin (bottom rows) was derived from phylogenetic trees and indicated as SC (southern China) and EC (eastern China). Three of the 4 reassortant genomes were also detected through environmental surveillance. M, matrix; NP, nucleoprotein; NS, nonstructural; PA, polymerase acidic; PB, polymerase basic.

#### HA and NA genes

The HA and NA genes of all H7N9 isolates from Guangdong Province we analyzed had high sequence identity (98.2%–99.7%) to viruses from other regions of China detected throughout 2013. However, the Guangdong Province isolates branched off and clustered with strains detected in Hong Kong during 2014 (Technical Appendix).

## Discussion

The outbreak of influenza A(H7N9) virus infection in humans in Guangdong Province that started in late 2013 coincided with the emergence of influenza A H7 viruses in LPMs as determined by environmental surveillance. The strong association between detection of human H7N9 cases and presence of H7 viruses in LPMs confirms that these market environments are sources of human exposure (*8*,*13*). A key question is how the virus has been spreading in China since the initial emergence in eastern China. A risk-mapping approach taking into account LPM locations, human population density, cropland irrigation, and meteorologic conditions listed central Guangdong Province as a high-risk area (defined as probability of the presence of influenza A[H7N9] viruses) (*14*). Little is known, however, about how H7N9 virus spreads and the prevalence of infection in commercial poultry flocks. 

The data from our study suggest sources of human infection in Guangdong Province are local. All sequenced viruses had evolved from the original strains through genetic reassortment with internal genes originating from influenza A(H9N2) viruses commonly found in southern China. The HA sequences of these viruses formed a distinct branch in phylogenetic trees, which indicates that reassortment occurred some time ago and suggests that local sources of virus transmission in Guangdong Province have not been detected by surveillance activities. However, available data are insufficient to determine how long influenza A(H7N9) viruses have been circulating in this region.

The results from environmental surveillance suggest a higher prevalence of H9N2 virus in Guangdong Province poultry environments than in poultry environments in other regions of China (*15*). Because live poultry is the primary source of H7N9 virus (*13*,*16*–*18*), the coexistence with H9N2 in the same susceptible population generates appropriate conditions for the emergence of novel reassortant variants such as those shown in this study. 

In summary, our findings indicate that multiple strains of H7N9 and H9N2 influenza viruses are circulating in poultry in Guangdong Province, creating an environment that is rich for reassortment of these viruses and that poses an ongoing risk for human infection. Continued and uncontrolled co-circulation of multiple avian and other influenza viruses that can infect humans pose a potential pandemic threat; increased animal surveillance and further study of the ecology of influenza viruses are essential.

Technical Appendix Phylogenetic trees of all gene segments of currently available influenza A(H7N9) viruses, showing locations of H7N9 and H9N2 viruses from Guangdong Province, China.
