# Supplementary material for: Circulation of Reassortant Influenza A(H7N9) Viruses in Poultry and Humans, Guangdong Province, China, 2013
Source: Emerg Infect Dis. 2014 Dec;20(12):2034–40. doi: 10.3201/eid2012.140765 (PMC4257815; doi:10.3201/eid2012.140765)
Supplement: Technical Appendix — Phylogenetic trees of all gene segments of currently available influenza A(H7N9) viruses, showing locations of H7N9 and H9N2 viruses from Guangdong Province, China. [file 14-0765-Techapp-s1.pdf]

Article DOI: http://dx.doi.org/10.3201/eid2012.140765

Circulation of Reassortant Influenza A(H7N9) Viruses in Poultry and Humans, Guangdong Province, China, 2013

Technical Appendix

Technical Appendix Figure (following pages). Phylogenetic trees of all gene segments of currently available influenza A(H7N9) viruses, showing locations of H7N9 and H9N2 viruses from Guangdong Province, China. Page 2, polymerase basic 2; page 3, polymerase basic 1; page 4, polymerase acidic; page 5, hemagglutinin; page 6, nucleoprotein; page 7, neuraminadase; page 8, matrix; page 9, nonstructural. Avian H9N2 viruses are shown in green, avian H9N2 viruses from Guangdong Province in blue, avian H7N9 viruses in orange, human H7N9 sequences from Guangdong Province in pink, and human H7N9 sequences from the main case cluster in 2013 in eastern China in red. Trees were generated by using PhyML (*1*).

Reference

1. Guindon S, Dufayard JF, Lefort V, Anisimova M, Hordijk W, Gascuel O. New algorithms and methods to estimate maximum-likelihood phylogenies: assessing the performance of PhyML 3.0. Syst Biol. 2010;59:307–21. PubMed http://dx.doi.org/10.1093/sysbio/syq010

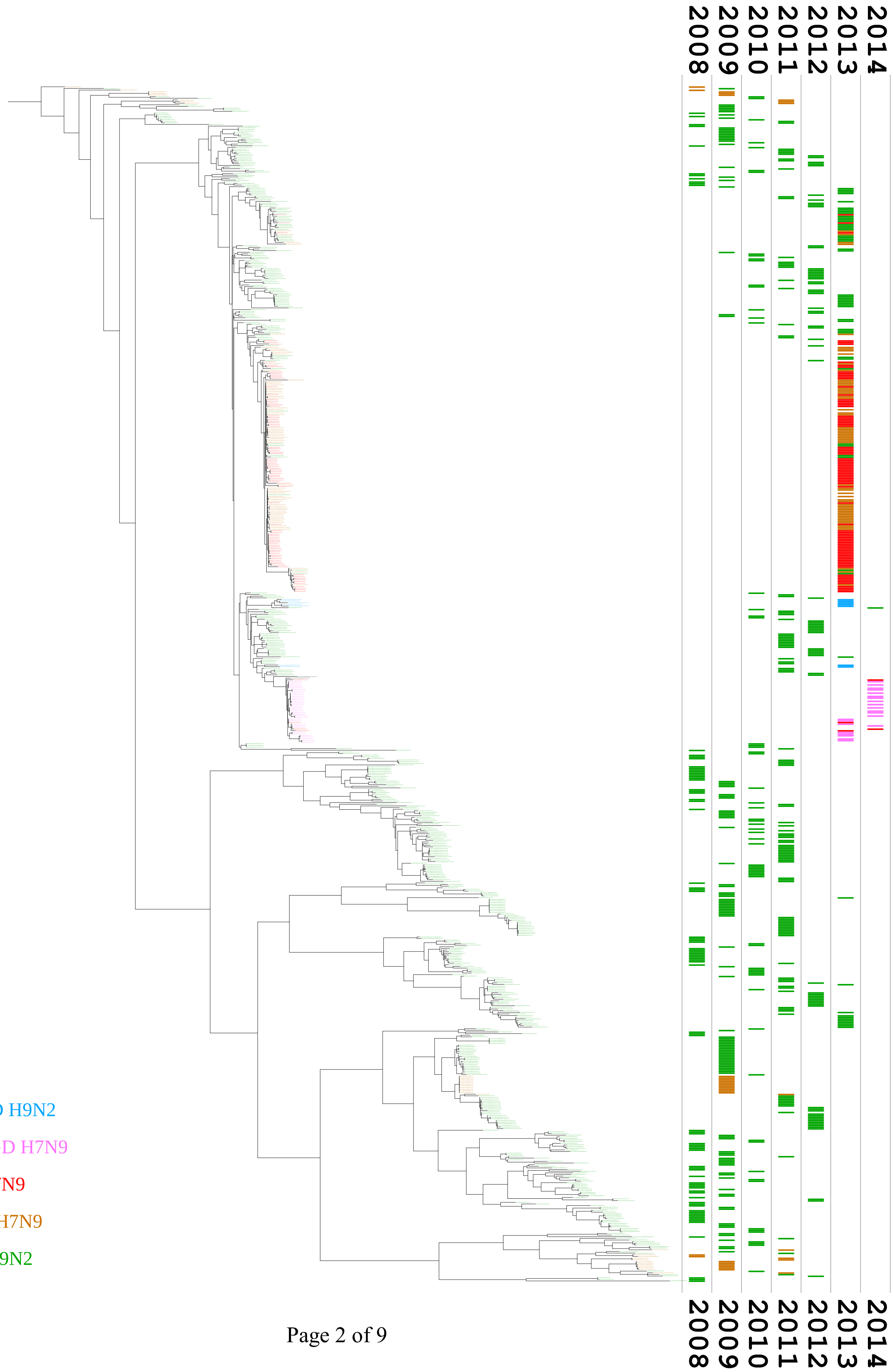

blue avian GD H9N2

pink human GD H7N9

red human H7N9

orange avian H7N9

green avian H9N2

PB2_150K

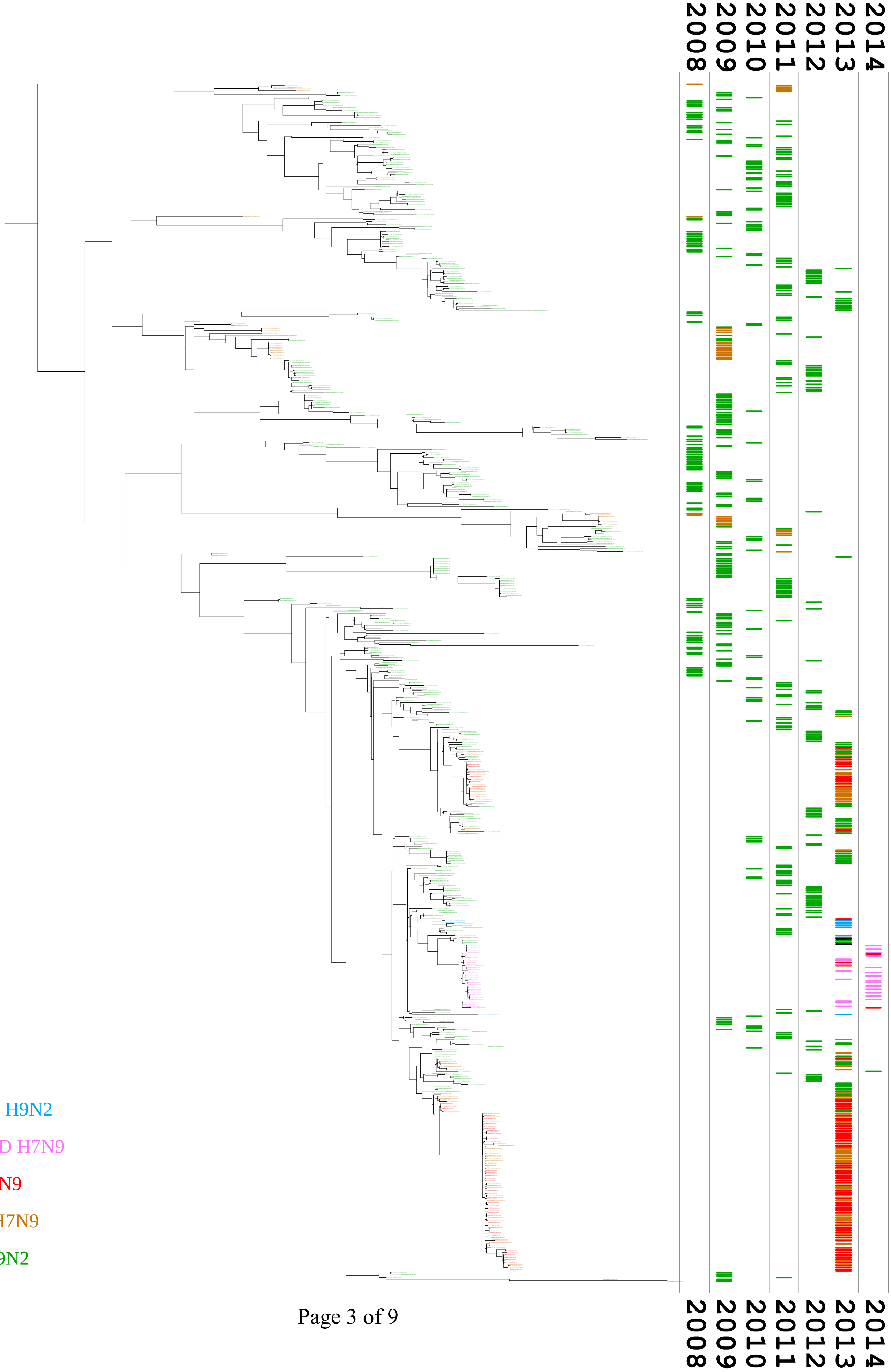

blue avian GD H9N2

pink human GD H7N9

red human H7N9

orange avian H7N9

green avian H9N2

PB1_150K

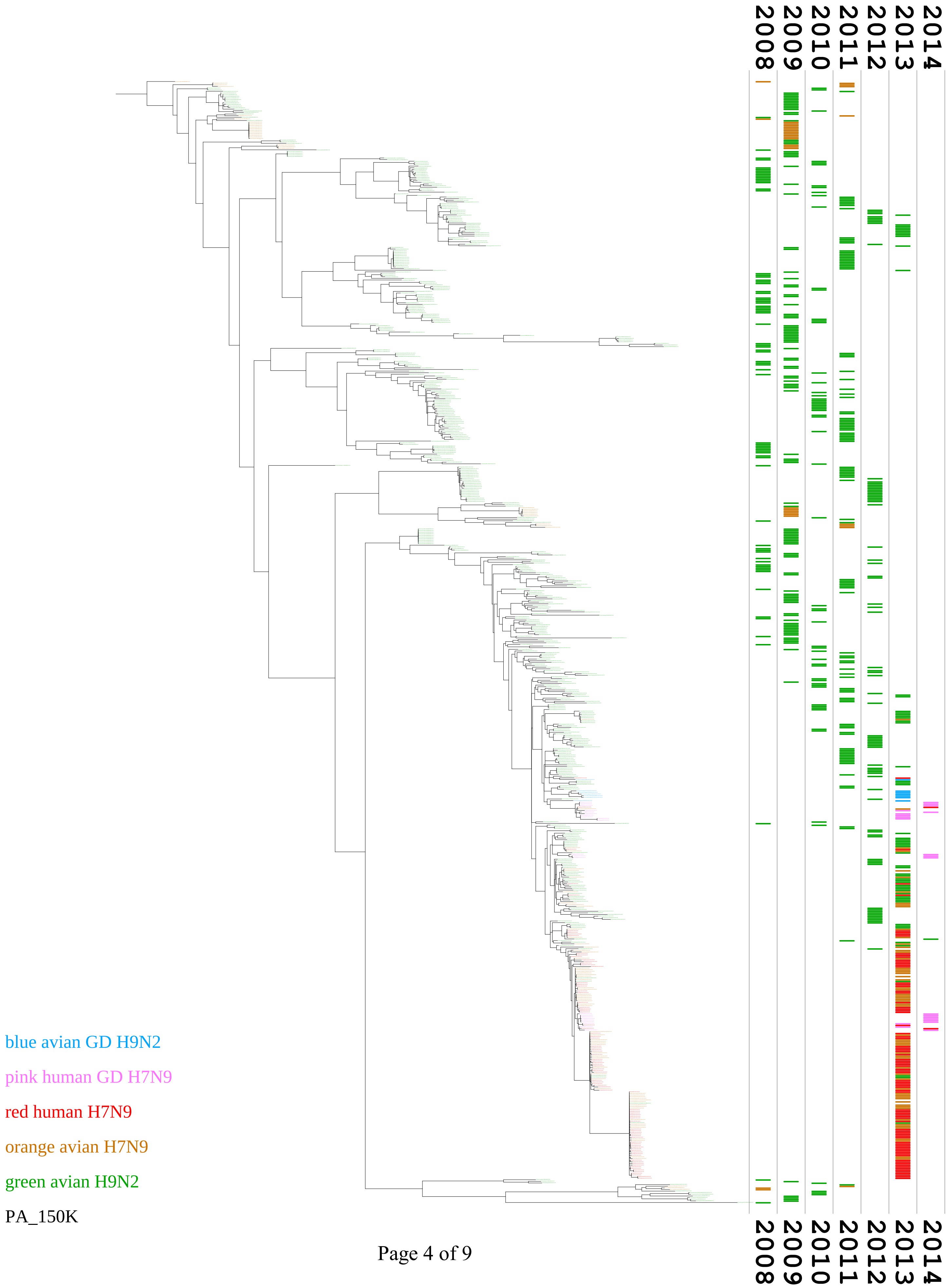
2008
2009
2010
2011
2012
2013
2014
blue avian GD H9N2
pink human GD H7N9
red human H7N9
orange avian H7N9
green avian H9N2
PA_150K

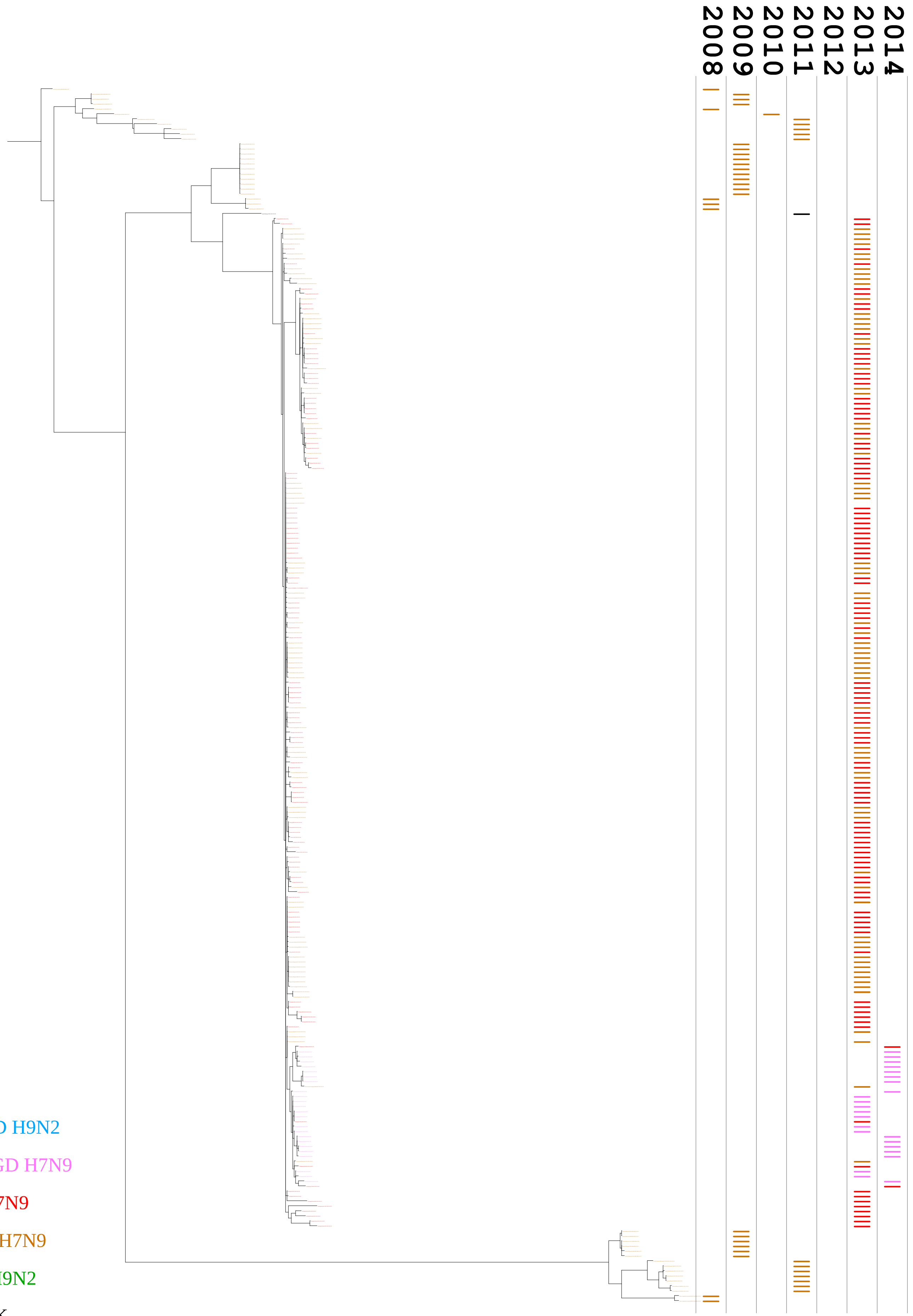

blue avian GD H9N2

pink human GD H7N9

red human H7N9

orange avian H7N9

green avian H9N2

HA_H7_150K

2008 2009 2010 2011 2012 2013 2014

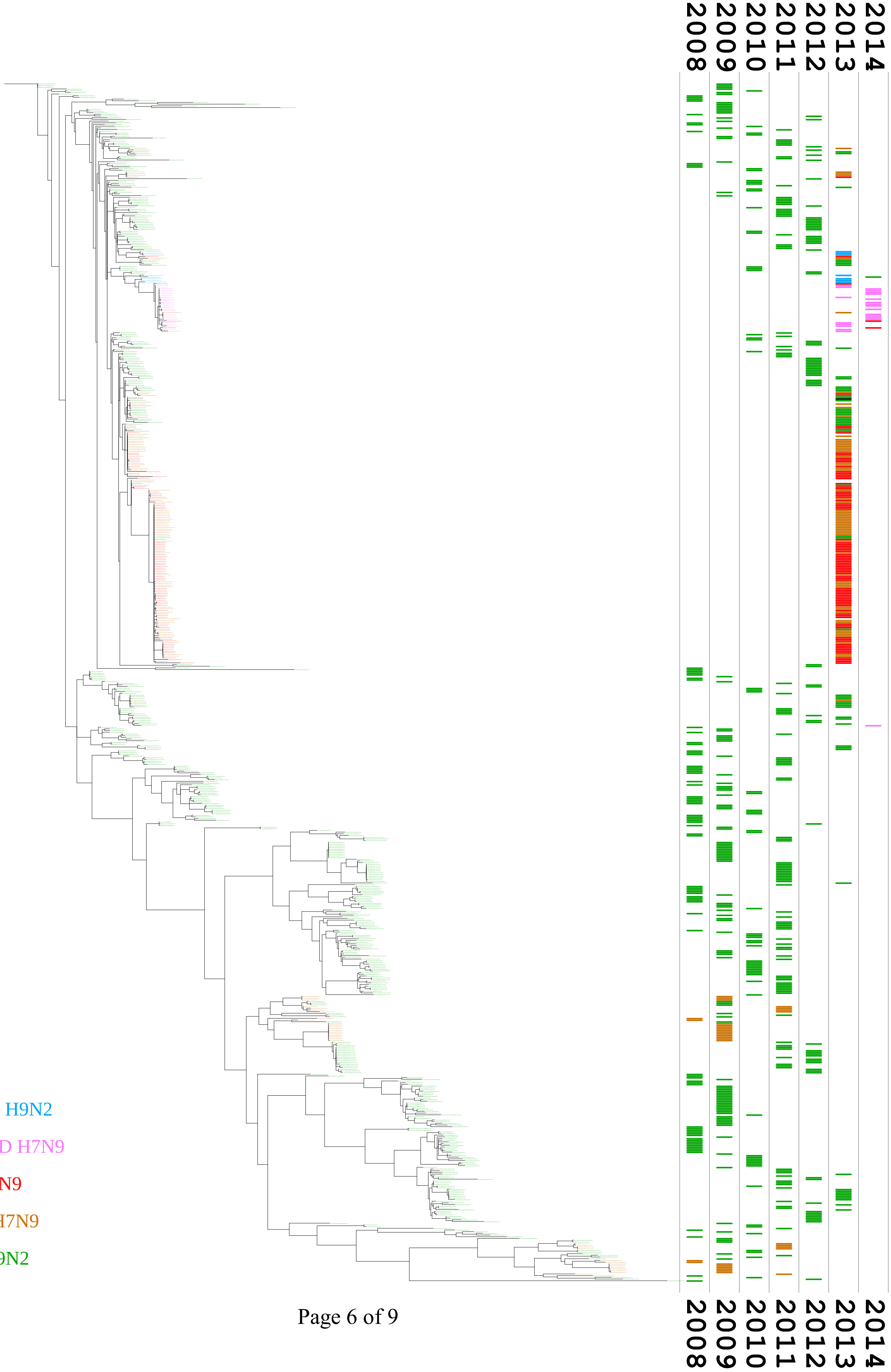

blue avian GD H9N2

pink human GD H7N9

red human H7N9

orange avian H7N9

green avian H9N2

NP_150K

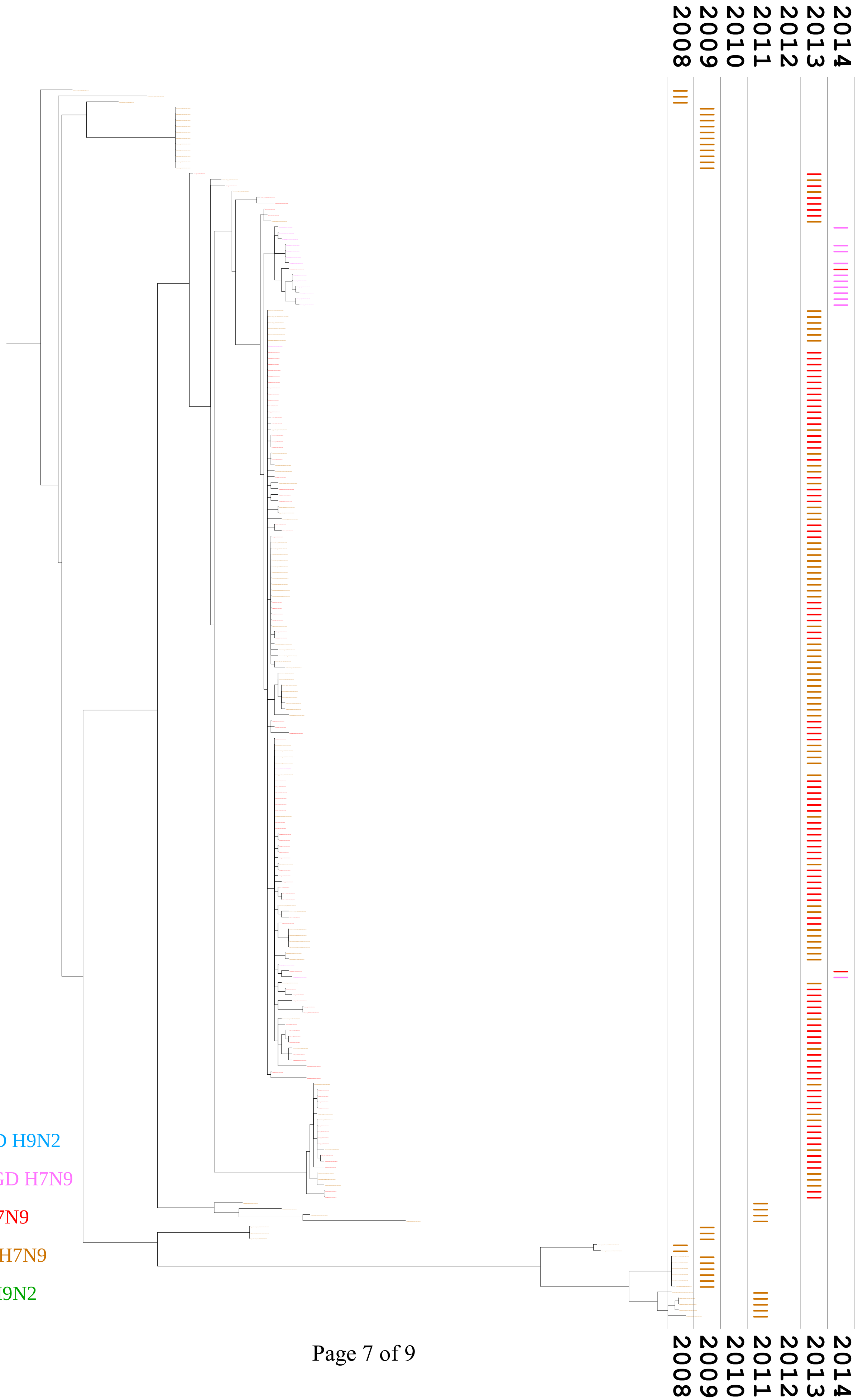

blue avian GD H9N2

pink human GD H7N9

red human H7N9

orange avian H7N9

green avian H9N2

NA_H7N9

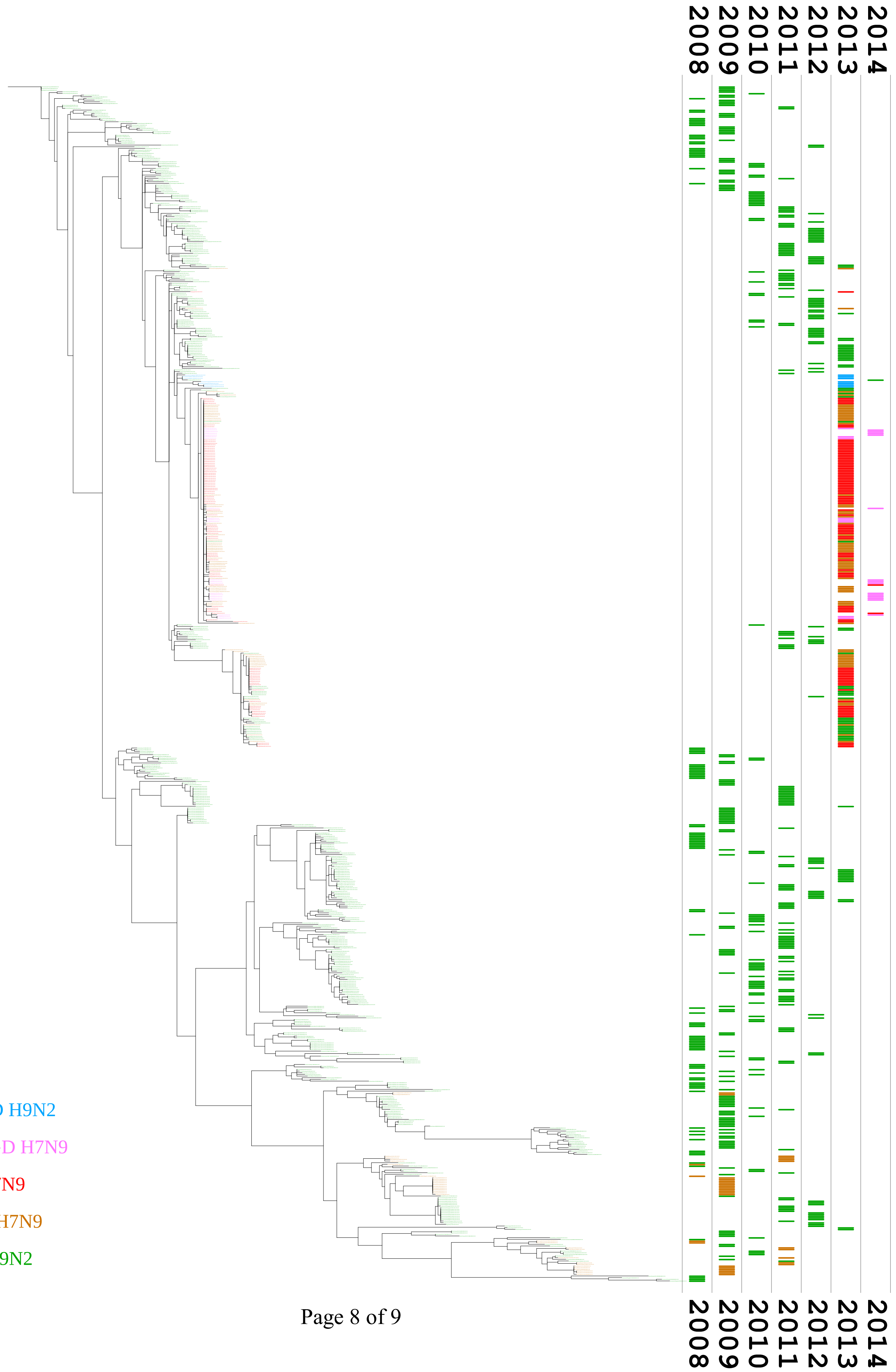

blue avian GD H9N2

pink human GD H7N9

red human H7N9

orange avian H7N9

green avian H9N2

MP

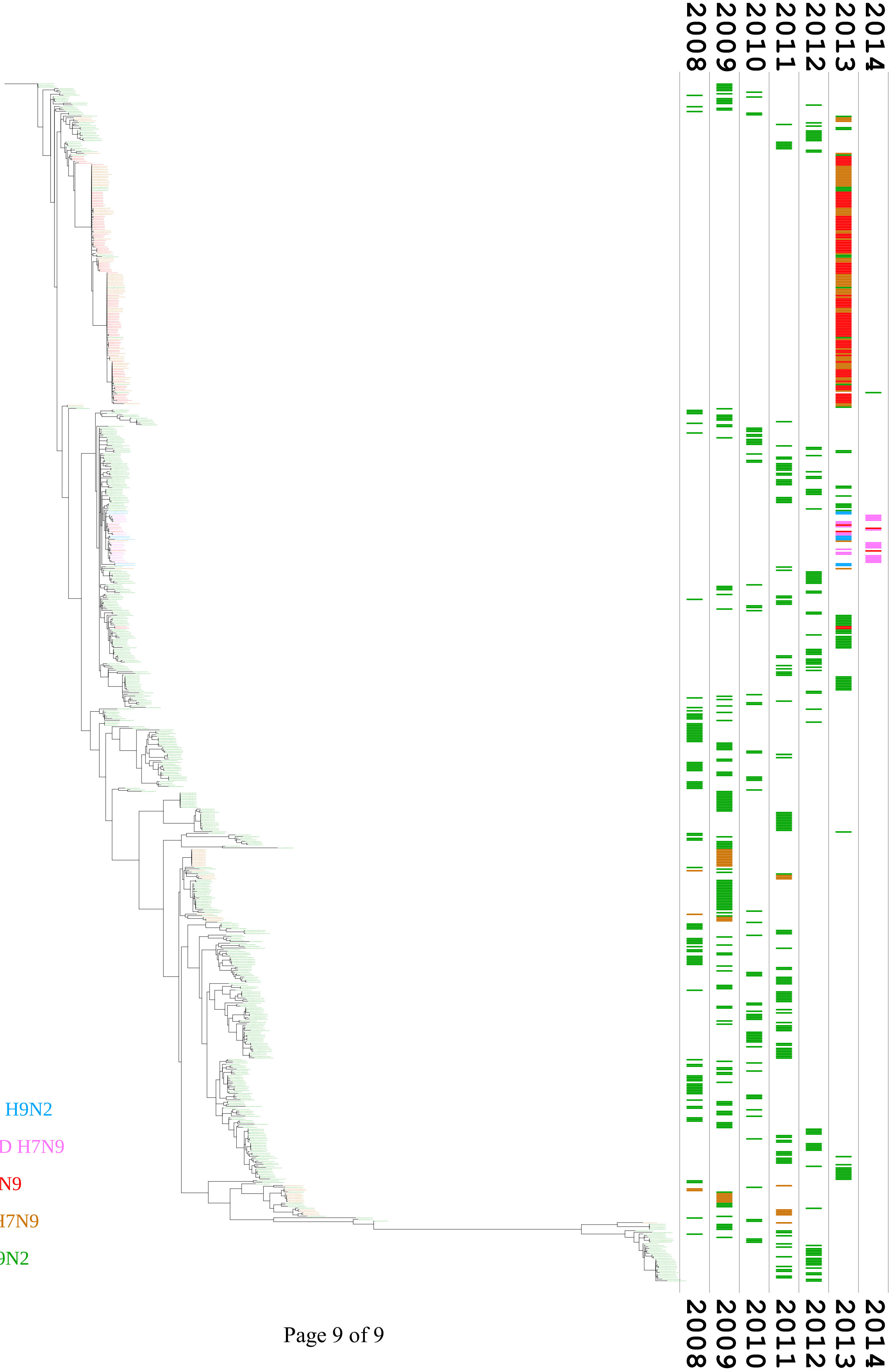

blue avian GD H9N2

pink human GD H7N9

red human H7N9

orange avian H7N9

green avian H9N2

NS
